# The impact of physical activity on social support among Chinese college students: the mediating role of physical self-esteem and the moderating effect of gender

**DOI:** 10.3389/fpsyg.2025.1695555

**Published:** 2025-12-01

**Authors:** Jingyi Wang, Baojian Wei, Shichang Cai, Hui Jiang

**Affiliations:** 1 Graduate School, Kunsan National University, Kunsan, Republic of Korea; 2 School of Nursing, Shandong First Medical University & Shandong Academy of Medical Sciences, Taian, Shandong, China; 3 School of Physical Education, Hengshui University, Hengyang, Hebei, China

**Keywords:** physical activity, social support, physical self-esteem, gender moderation, college students, mediation effect, moderated mediation model

## Abstract

**Objective:**

This study aimed to examine the mechanisms through which physical activity influences social support among Chinese college students by constructing a moderated mediation model, with physical self-esteem as a mediator and gender as a moderator.

**Methods:**

A total of 667 undergraduates and postgraduates (327 males, 340 females) from a comprehensive university in Jiangsu Province were recruited using cluster convenience sampling. Physical activity (PARS-3), physical self-esteem (College Students’ Physical Self-Esteem Scale), and social support (Adolescent Social Support Rating Scale) were measured via structured questionnaires. Multi-level statistical analyses were conducted using the SPSS PROCESS macro to test mediation and moderated mediation effects.

**Results:**

(1) Physical activity significantly and positively predicted social support (*β* = 0.06, *p* < 0.001); (2) Physical self-esteem partially mediated this relationship, with an indirect effect of 0.02, accounting for 33.33% of the total effect; (3) Gender significantly moderated the pathway between physical activity and social support. Among females, the effect was significantly positive (*β* = 0.12, *p* < 0.001), whereas among males, the effect was negative (*β* = −0.05, *p* < 0.001).

**Conclusion:**

These findings demonstrate that physical activity enhances social support both directly and indirectly through increased physical self-esteem, while gender differences shape the strength and direction of this relationship. Specifically, physical activity benefits female students’ social support but may reduce that of male students, possibly due to the competitive orientation of their sports participation. The study extends social integration and self-perception theories and highlights the importance of gender-sensitive interventions in university-based physical activity and mental health programs.

## Introduction

The college stage represents a critical transitional period for the reconstruction of personal social networks and psychological adaptation. During this time, students not only face considerable academic pressure but also encounter social challenges associated with integrating into new communities, making them a high-risk group for psychological distress (Leary et al., 1995). Within the highly competitive environment of higher education in China, multiple stressors—such as academic workload, interpersonal competition, and anxiety about future prospects—interact to potentially reduce students’ perceived social support, thereby exacerbating feelings of loneliness. From an academic perspective, the central role of social support in individual adaptation and development has been widely established (Cohen and Wills, 1985). The classic stress-buffering model posits that social support, through providing emotional comfort, informational assistance, and instrumental help, can effectively mitigate the adverse effects of stressful events on both psychological and physiological health. Recent meta-analyses further confirm that high-quality social support systems are significantly and positively associated with academic achievement, mental health (reflected in lower levels of depression and anxiety symptoms), and overall life satisfaction among college students. Therefore, identifying effective strategies to enhance social support has become an important focus of university-based mental health education. In this context, physical activity—an intervention characterized by low cost, ease of promotion, and absence of pharmaceutical side effects—has increasingly drawn scholarly attention for its potential value in improving social support. Although existing research has provided preliminary evidence of a positive association between physical activity and social support (Bruner et al., 2014), the specific pathways of this effect and its contextual boundaries remain unclear. More specifically, through which psychological mechanisms does physical activity translate into enhanced social support? Do such effects vary across different demographic groups? These critical theoretical questions warrant further investigation.

Previous studies, employing diverse methodologies, have consistently demonstrated a significant positive association between physical activity and social support (Lonsdale et al., 2013). This link suggests that increases in the frequency, intensity, and duration of physical activity are often accompanied by concurrent enhancements in perceived social support. From an intervention perspective, the strong operability, low cost, and non-pharmacological nature of physical activity make it a promising approach to expanding individuals’ social support networks. Participation in team-based sports, in particular, has shown pronounced benefits in fostering social connectedness, a sense of belonging, and peer support among adolescents (Lonsdale et al., 2013). Moreover, a meta-analysis covering 50 high-quality studies confirmed that physical activity interventions significantly reduce loneliness—an emotional state closely linked to a lack of social support in adults (Hagan et al., 2014). These empirical findings support the explanatory framework of social integration theory, which emphasizes that shared participation in goal-directed collective activities promotes the formation of social bonds and the expansion of social networks (Thoits, 1983). Sports and group exercise, as prototypical structured interaction settings, provide sustained opportunities for social contact and foster emotional connections through cooperative goal attainment and mutual assistance, thereby directly enhancing both the size and quality of social support networks. Longitudinal research on university students by further substantiates this mechanism: students who maintained regular participation in physical activity reported higher levels of perceived social support and lower levels of social isolation over time, confirming the sustained positive impact of physical activity on social support. Based on the above theoretical and empirical foundations, this study proposes its first hypothesis:

*H1*: Participation in physical activity is significantly and positively correlated with perceived social support.

Physical self-esteem, defined as an individual’s overall subjective evaluation of their physiological characteristics, physical functioning, and athletic performance, has been widely recognized as an important psychological outcome of physical activity (Cash and Smolak, 2011; Myers, 2012). A meta-analysis by Ma et al. (2020), building upon the classic conclusions of Sonstroem and Morgan (1989) and Spence et al. (2005) incorporating 37 experimental and quasi-experimental studies published between 2000 and 2019, confirmed the robust positive effects of physical activity on body image and physical self-esteem. Moderate-intensity, regular physical activity performed three to five times per week (e.g., aerobic exercise, strength training) was found to increase physical self-esteem scores by approximately 15–20% (Bassett-Gunter et al., 2017). Notably, the study also revealed variation in effects across exercise types: mind–body practices such as yoga and Pilates, by enhancing mind–body connection and reducing social comparison, significantly improved body acceptance and positive body image—effects that in some cases exceeded those of traditional competitive sports (Alleva et al., 2023). The association between physical self-esteem and social support can be explained through the micro-mechanisms of social interaction. Higher physical self-esteem indicates a more positive self-perception of one’s body image, which directly influences behavior patterns and emotional experiences in social contexts. According to the social physique anxiety model (Leary et al., 1995), self-perceptions of physical appearance significantly regulate social anxiety levels: individuals with low evaluations of their physique tend to anticipate negative judgments from others, experience “evaluative anxiety,” and avoid social interactions; conversely, those with high physical self-esteem are less likely to experience such anxiety and exhibit greater social initiative. This mechanism has been confirmed in longitudinal studies of adolescents. For instance, Rodgers et al. (2021), in a three-year study of 1,200 adolescents aged 12–16, found that improvements in positive body image significantly predicted higher peer relationship quality (*β* = 0.28, *p* < 0.01) and lower social anxiety (*β* = −0.22, *p* < 0.05) in the following year, providing dynamic evidence of a causal link between physical self-esteem and social support. Self-perception theory offers an integrative explanatory framework for understanding the mediating role of physical self-esteem (Bem, 1972). The theory posits that individuals infer internal attitudes and traits through observing their own behaviors and the outcomes thereof. When students engage in physical activity and observe improvements in physical strength, appearance, or athletic ability, these positive outcomes foster a self-perception of “having a good physical condition,” thereby enhancing physical self-esteem. This cognitive restructuring, triggered by overt behavior, reduces social apprehension (e.g., fear of negative body evaluation), increases willingness to engage in group activities or initiate interpersonal interactions, and ultimately facilitates the acquisition of social support resources such as emotional companionship and instrumental help. Based on this theoretical reasoning and empirical evidence, this study proposes the following hypotheses:

*H2*: Participation in physical activity positively predicts levels of physical self-esteem.

*H3*: Physical self-esteem mediates the relationship between physical activity and social support.

Finally, this study hypothesizes that gender may play a moderating role in the relationship between physical activity and social support. Extensive empirical evidence supports gender differences in psychosocial outcomes (Hyde, 2005). Specifically, research has demonstrated systematic gender differences in both the structural characteristics (e.g., network size, heterogeneity) and functional attributes (e.g., patterns of obtaining emotional and instrumental support) of social networks (Rose and Rudolph, 2006). Similarly, gender differences in sports participation are well-documented—from participation frequency and activity preferences (e.g., women favoring lower-intensity activities such as yoga or dance, men preferring ball games or strength training) to motivational orientations (e.g., women emphasizing health and social connection, men focusing on competition and physical display) (Guthold et al., 2018). Given that gender independently influences both physical activity participation and the formation of social support, it is logically reasonable to posit a moderating role of gender in their relationship (Eagly, 2013; Eagly and Wood, 2012; Van Lange et al., 2012). Within the framework of social role theory, cultural norms assign different behavioral expectations to men and women. For female college students, physical activity is often framed as a means of health maintenance, social bonding, and aesthetic improvement, aligning closely with socially prescribed female roles emphasizing affiliation and cooperation. Consequently, frequent participation in such activities not only increases opportunities for interpersonal interaction but also fosters the accumulation of social support resources through a “social connection enhancement → support resource accumulation” pathway. In contrast, male gender roles emphasize dominance, competitiveness, and physical prowess. Although participation in competitive sports aligns with the “competitiveness” characteristic of male roles, interaction patterns that overly emphasize win–loss outcomes may weaken the depth of emotional bonding. In high-intensity competitive environments, the “winner–loser” dichotomy, combined with time constraints imposed by intensive training, may to some extent hinder the development of intimate supportive relationships. This potential conflict between role expectations suggests that, among men, the relationship between physical activity and social support may be nonlinear: once participation intensity exceeds a certain threshold, behaviors intended to reinforce social status may inadvertently erode the foundation of broad social support. Based on the above reasoning, the fourth hypothesis is proposed:

*H4*: Gender moderates the relationship between physical activity and social support, with the association being stronger among females and potentially weaker or negative among males.

**Figure 1:**
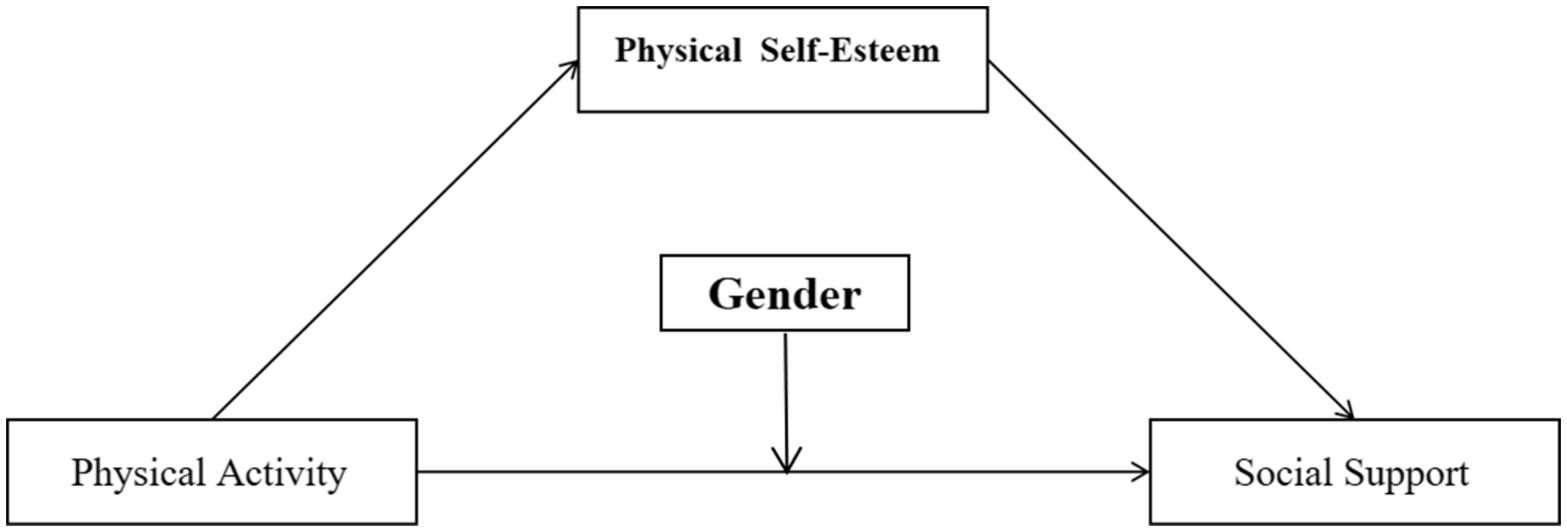
The mediating moderating hypothesis model of the impact of physical activity on social support.

The present study aims to address this research gap by constructing a moderated mediation model (Figure 1) to systematically examine the pathways through which physical activity affects social support among Chinese college students. Specifically, physical self-esteem is introduced as a mediator to reveal the underlying psychological mechanism, while gender is incorporated as a moderator to test for differential effects and boundary conditions across gender groups. Through theoretical modeling and empirical testing, the study seeks to provide new theoretical underpinnings and practical approaches for interventions aimed at enhancing social support.

## Methods

### Participants

This study adopted a cross-sectional design and employed a convenience sampling approach, using intact natural administrative classes from a comprehensive university in Jiangsu Province as the sampling units. At the initial stage, a statistical power analysis was conducted using G*Power software (version 3.1) with the parameters set at *α* = 0.05, *β* = 0.20, and effect size d = 0.15, yielding a predetermined sample size of 600 participants. Accordingly, 600 questionnaires were planned for distribution; however, during implementation, the distribution was expanded to 700 to further enhance statistical power. Data collection was conducted via an electronic questionnaire. Prior to administration, the research team organized offline briefing sessions for all potential participants, during which the study objectives, procedures, and ethical guidelines were systematically explained. Questions were addressed on-site, and written informed consent forms were signed in paper format to ensure the informed consent process was standardized and traceable. Operationally, upon obtaining formal written authorization from the class advisors, participants were instructed to complete the anonymized electronic questionnaire in an independent setting. Throughout the process, collection of personally identifiable information (e.g., student ID, national ID number) was strictly prohibited to safeguard data privacy. The study was conducted in full compliance with the ethical principles outlined in the Declaration of Helsinki for research involving human participants and was approved by the Ethics Committee of Hengshui University.

For data quality control, standardized quality assurance procedures were rigorously implemented. Exclusion criteria included: (1) random response bias (missing data on >20% of key items); (2) abnormal response time (completion time <120 s or >1,800 s); (3) patterned responding (cyclical repetition across 12 consecutive items); (4) excessive option repetition (selecting the same option ≥8 consecutive times and accounting for ≥30% of total responses). Following independent double-check verification (Kappa coefficient = 0.82), a total of 667 valid questionnaires were retained, resulting in a response rate of 95.29%. Among the valid respondents, 340 were female (50.97%) and 327 were male (49.03%). Gender distribution met the assumption of normality according to the Shapiro–Wilk test (W = 0.98, *p* = 0.12).

Participant selection strictly adhered to five inclusion criteria: (1) Educational level: Full-time, currently enrolled undergraduate or graduate students in regular higher education. Referring to the research of Fox and Lindwall, 2014, excluding students majoring in sports or psychology to avoid potential bias from professional training in physical or psychological domains; (2) Cognitive capacity: Clear consciousness and no reading difficulties; exclusion of individuals with a history of psychiatric disorders (e.g., depression, anxiety) or currently undergoing related treatment, screened via self-report questionnaires and verified against university counseling center records; (3) Dual-channel informed consent: Confirmation via both electronic signature and handwritten paper signature to ensure voluntary participation and awareness of the right to withdraw at any stage; (4) Dual authorization for compliance: Co-signature of the “Research Participation Confirmation Form” by both the academic affairs administrator (associate dean of the faculty) and the participant; (5) Physical health: Absence of clinically diagnosed chronic diseases (e.g., hypertension, diabetes) or acute-phase illnesses, verified using medical examination records from the university hospital. The operational process followed a three-tier review mechanism: First-tier: Preliminary eligibility screening by the class advisor (verification of basic student information and health status); Second-tier: On-site verification by the principal investigator (assessment of questionnaire completeness and response time plausibility); Third-tier: Back-end logical consistency checks using SPSS 26.0. This procedural framework formed a complete ethical review loop, ensuring that the research process adhered to both the Declaration of Helsinki and the Chinese “Ethical Review Measures for Biomedical Research Involving Humans” (Ekeland et al., 2002).

### Measures

#### Physical activity

Physical activity was assessed using the Physical Activity Rating Scale-3 (PARS-3; Rees and Freeman, 2007), a widely applied instrument for evaluating exercise behavior in adolescents and young adults (McAuley et al., 2000). The scale comprises three items measuring exercise intensity (1–5 points; 1 = “light activity,” 5 = “vigorous activity”), frequency (1–5 points; 1 = “once a month or less,” 5 = “daily or more”), and duration (0–4 points; 0 = “<10 min,” 4 = “≥60 min”). A composite score was calculated using the formula: Physical Activity Score = Intensity × Frequency × Duration. The possible range is 0–100, with higher scores indicating greater physical activity engagement. In this study, Cronbach’s *α* values for the scale were 0.812 (pilot test) and 0.843 (main survey), demonstrating good internal consistency.

### Physical self-esteem

Physical self-esteem was measured using the College Students’ Physical Self-Esteem Scale (Marsh et al., 1994; Sonstroem and Morgan, 1989; Fox and Lindwall, 2014), developed based on Fox’s theoretical framework. The instrument consists of 30 items (including 16 reverse-scored items) covering one global dimension—Physical Self-Worth—and four subscales: Sport Competence, Physical Condition, Physical Attractiveness, and Physical Fitness. Items are rated on a 4-point Likert scale (1 = “not at all true” to 4 = “completely true”), with higher scores reflecting higher physical self-esteem. In the present study, Cronbach’s *α* for the total scale was 0.837, while subscale α values ranged from 0.721 to 0.803, meeting psychometric reliability standards.

### Social support

Social support was assessed using the Adolescent Social Support Rating Scale (Zimet et al., 1988; Lakey and Cohen, 2000; Cutrona and Russell, 1987), adapted from Zimet’s et al., 1988 model for Chinese adolescents and young adults. The instrument contains 17 items across three dimensions: Subjective Support (5 items), Objective Support (6 items), and Support Utilization (6 items). Each item is rated on a 5-point Likert scale (1 = “not at all true” to 5 = “completely true”), with a total score ranging from 17 to 85. Higher scores indicate greater perceived social support. In this study, Cronbach’s α values for the total scale were 0.901 (pilot test) and 0.912 (main survey), with subscale α values ranging from 0.823 to 0.885, indicating excellent reliability. The total score range of this scale is 17–85 points. In order to facilitate cross variable comparison and horizontal comparison with the Physical Self Esteem Scale (4-point scoring, mean range 1–4) and the Standardized Physical Activity Score (1–5 points), this study converted the original total score to the mean score (conversion formula: mean score = original total score/17), and the converted mean range is 1–5 points. The subsequent tables (Tables 1, 2) present the converted average scores to visually reflect the relative levels of each variable.

**Table 1:** Descriptive statistics and correlation matrix.

Variable	*M*	SD	Gender	Physical activity	Physical self-esteem	Social support
Gender	1.49	0.50	1			
Physical activity	2.43	2.48	−0.01	1		
Physical self-esteem	2.26	0.48	0.11**	0.36**	1	
Social support	3.43	0.73	−0.05	0.21**	0.24**	1

NM = mean, SD = standard deviation; Gender coded as female = 1, male = 2; *N* = 667; **p < 0.01, *p* < 0.001.

**Table 2:** Mediating effect test of physical self-esteem.

Predictive variables	Regression equation 1: physical self-esteem			Regression equation 2: social support			Regression equation 3: social support		
	*α*	SE	*t*	*α*	SE	*t*	*α*	SE	*t*
Physical activity	0.07***	0.01	9.98	0.06***	0.01	5.71	0.04**	0.01	3.72
Physical self-esteem							0.28***	0.06	4.66
*R*	0.36			0.22			0.28		
*R* ^2^	0.13			0.05			0.08		
*F*	99.56***			32.59***			27.70***		

**p < 0.01, ***p < 0.001.

### Statistical analysis

This study employed IBM SPSS Statistics 27.0 and its extension macro PROCESS v4.1 to conduct a multi-stage statistical analysis (Hayes, 2018), with the aim of examining the pathways through which physical activity influences social support among college students, and to further explore the mediating role of physical self-esteem as well as the moderating effect of gender. The specific analytical procedure was as follows: First, to assess the risk of common method bias, Harman’s single-factor test was performed on the questionnaire data using an unrotated principal component analysis. If the variance explained by the first common factor did not exceed 40% of the total variance, it was deemed that no significant common method bias existed. Second, descriptive statistics for each variable—mean (M) and standard deviation (SD)—and Pearson’s correlation coefficients (r) were computed to preliminarily examine the bivariate relationships among physical activity, physical self-esteem, social support, and gender. Third, the bias-corrected bootstrap method (with 5,000 resamples) was applied to test the mediating effect of physical self-esteem on the relationship between physical activity and social support using Model 4 of the PROCESS macro. The evaluation criteria included whether the 95% confidence interval (CI) of the indirect effect excluded zero and the proportion of the total effect accounted for by the mediating effect. Next, following the analytical framework for moderated mediation models, Model 5 of the PROCESS macro was used to investigate the moderating role of gender in the relationship between physical activity and social support. To avoid potential multicollinearity, all continuous variables were mean-centered prior to constructing the interaction term. If the interaction term between physical activity and gender was statistically significant and the direction of the moderating effect was consistent with the hypothesis, this would indicate that gender significantly moderated the main effect pathway. Finally, to illustrate the specific nature of the moderating effect, simple slope analyses were conducted for significant interactions, and interaction plots were generated. The Johnson–Neyman technique was employed to identify regions of significance for the effect, thus pinpointing the threshold and trend changes of physical activity’s impact at different gender and variable levels, revealing the critical points of gender moderation. Throughout the analysis, the statistical procedure adhered strictly to the principle of combining confidence interval methods with non-parametric testing to ensure the robustness of causal pathway testing. The significance threshold was uniformly set at *α* = 0.05, and all confidence intervals were two-sided at the 95% level.

## Results and analysis

### Common method bias test

Given that all variables in this study were measured via participants’ self-reports on the Wenjuanxing platform, there was potential for common method bias to affect the results. To assess this possibility, Harman’s single-factor test was conducted on all measurement data using an unrotated exploratory factor analysis (Han and Zhao, 2025). The analysis extracted nine factors with eigenvalues greater than 1, with the first factor accounting for 25.03% of the total variance—below the critical threshold of 40%. This result indicates that common method bias was not a significant concern, thereby supporting the reliability and validity of the study’s findings.

### Correlations among variables

A correlation analysis was conducted for four variables: physical activity, physical self-esteem, social support, and gender. The results revealed that physical activity was significantly and positively correlated with both physical self-esteem and social support, indicating that higher levels of physical activity are closely associated with enhanced physical self-esteem and increased social support. The correlation between physical activity and gender was not statistically significant. Physical self-esteem demonstrated significant positive correlations with both social support and gender, suggesting that greater physical self-esteem is associated with higher levels of social support and certain gender-related differences. In contrast, the relationship between gender and social support was not statistically significant (see Table 1 for detailed results).

### Relationship between physical activity and social support: mediating role of physical self-esteem

Physical activity was entered as the independent variable, social support as the dependent variable, and physical self-esteem as the mediator. Based on the frameworks of Hayes (2013) and Wen and Ye (2014), PROCESS macro Model 4 was employed to test the mediating effect. The results (Table 2) showed that: Physical activity significantly and positively predicted physical self-esteem (*β* = 0.07, *p* < 0.001). Physical activity significantly predicted social support (*β* = 0.06, *p* < 0.001), confirming the existence of the direct effect. When both physical activity and physical self-esteem were entered into the regression equation, both remained significant positive predictors of social support, supporting the hypothesized mediation model.

**Figure 2:**
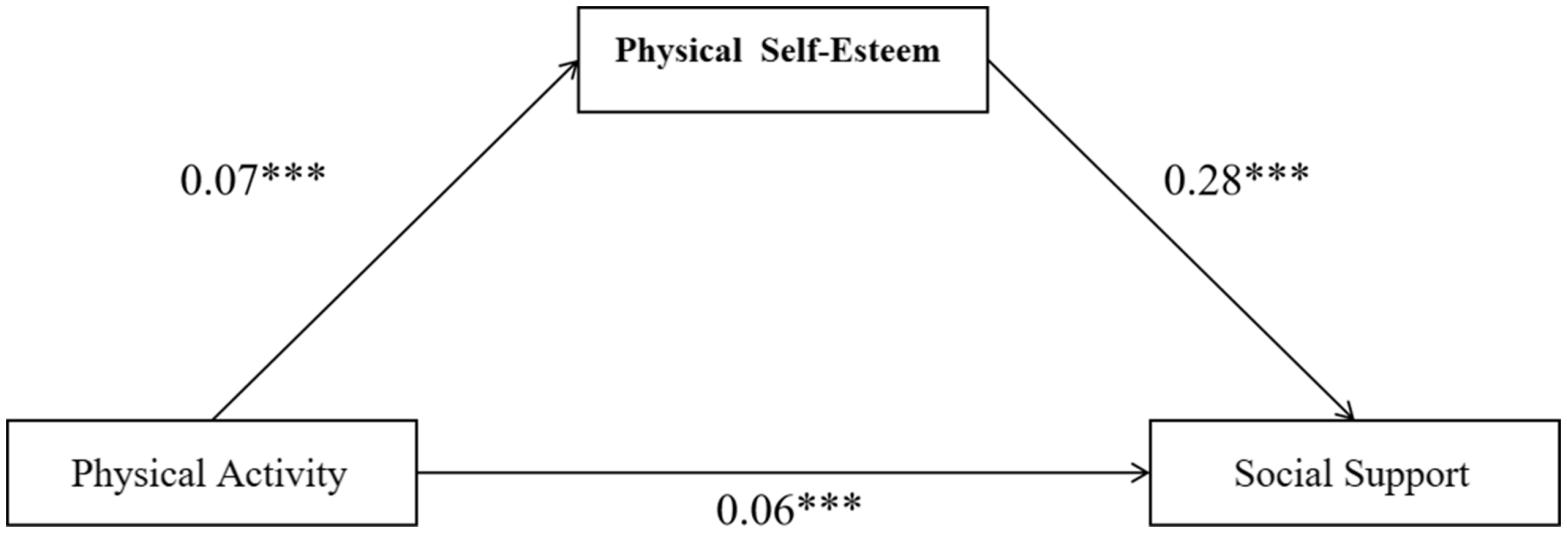
Model of physical self esteem as a mediating role between physical activity and social support.

A bias-corrected percentile bootstrap method was further employed for testing (Ren et al., 2025), and the relevant results are presented in Table 3. The findings indicated that the total effect of physical activity on social support was 0.06, with a bootstrap standard error (Boot SE) of 0.01 and *p* < 0.001. The corresponding 95% confidence interval (CI) was [0.04, 0.09], which did not include zero, thereby confirming the significance of the total effect. In addition, the indirect effect of physical activity on social support through physical self-esteem was 0.02, with a Boot SE of 0.01 and p < 0.001. The corresponding 95% CI was [0.01, 0.03], which likewise excluded zero, indicating that the mediating role of physical self-esteem in the relationship between physical activity and social support was statistically significant (see Figure 2). Further calculation showed that the mediating effect accounted for 33.33% of the total effect.

**Table 3:** Bootstrap mediation analysis.

Effect type	Path	Effect	SE	LLCI	ULCI	% of total effect
Direct effect	Physical activity → Social support	0.04***	0.01	0.02	0.07	66.67%
Indirect effect	Physical activity → Physical self-esteem → Social support	0.02***	0.01	0.01	0.03	33.33%
Total effect	(Direct + Indirect)	0.06***	0.01	0.04	0.09	100%

**p < 0.01, ***p < 0.001.

### Relationship between physical activity and social support: mediating role of physical self-esteem and moderating role of gender

This study adopted Hayes’ framework for testing moderated mediation effects, employing PROCESS macro version 4.1 (Model 5) for parameter estimation (Hayes, 2015). All continuous core variables were standardized (mean-centered) to eliminate scale differences prior to analysis. The regression model was constructed with physical activity as the independent variable, social support as the dependent variable, physical self-esteem as the mediator, and gender as the moderator. A bootstrapping procedure with 5,000 resamples was applied to construct 95% confidence intervals (CIs) for significance testing. The regression results for the moderated mediation model are presented in Table 4. The testing of a moderated mediation model requires estimation of the following:(a) the predictive effect of physical activity on physical self-esteem; (b) the total effect of physical activity on social support; (c) the main effect of physical activity on social support and the interaction effect between physical activity and gender. A significant moderated mediation effect can be established if all three conditions are met (Ding et al., 2024; Carron et al., 1996; Preacher and Hayes, 2008; Stice and Shaw, 2002).

**Table 4:** Simple slope analysis by gender.

Moderator	Effect	SE	*t*	LLCI	ULCI
Female	0.12***	0.01	8.17	0.09	0.15
Male	−0.05***	0.02	−3.22	−0.08	−0.02

***p < 0.001.

As shown in Table 5, the results indicated that physical activity significantly and positively predicted physical self-esteem (*β* = 0.07, *p* < 0.001), satisfying condition (a). Physical activity also exerted a significant positive effect on social support (*β* = 0.29, *p* < 0.01), meeting condition (b). When the interaction term of physical activity and gender was entered into the regression equation, the main effect of physical activity on social support remained significant (*β* = 0.29, *p* < 0.01), and the interaction term was also significant (*β* = −0.17, *t* = −8.17, *p* < 0.001). This finding indicates that gender significantly moderates the relationship between physical activity and social support, thereby confirming the hypothesized moderated mediation effect. Moreover, all variance inflation factors (VIFs) for the predictors were ≤ 1.2, suggesting no multicollinearity issues.

**Table 5:** Regression analysis of moderated mediation effects.

Predictor	Eq.1: physical self-esteem					Eq.2: social support					Eq.3: social support				
	*β*	SE	*t*	LLCI	ULCI	*β*	SE	*t*	LLCI	ULCI	*β*	SE	*t*	LLCI	ULCI
Physical activity	0.07***	0.01	9.98	0.06	0.08	0.06***	0.01	5.71	0.04	0.09	0.29***	0.03	8.98	0.23	0.35
Physical self-esteem											0.33***	0.06	5.7	0.22	0.45
Gender											0.30***	0.07	4.18	0.16	0.45
Physical activity × gender											−0.17***	0.02	−8.17	−0.21	−0.13
*R* ^2^	0.13					0.05					0.17				
*F*	99.56					32.59					33.06				

**p < 0.01, ***p < 0.001.

**Figure 3:**
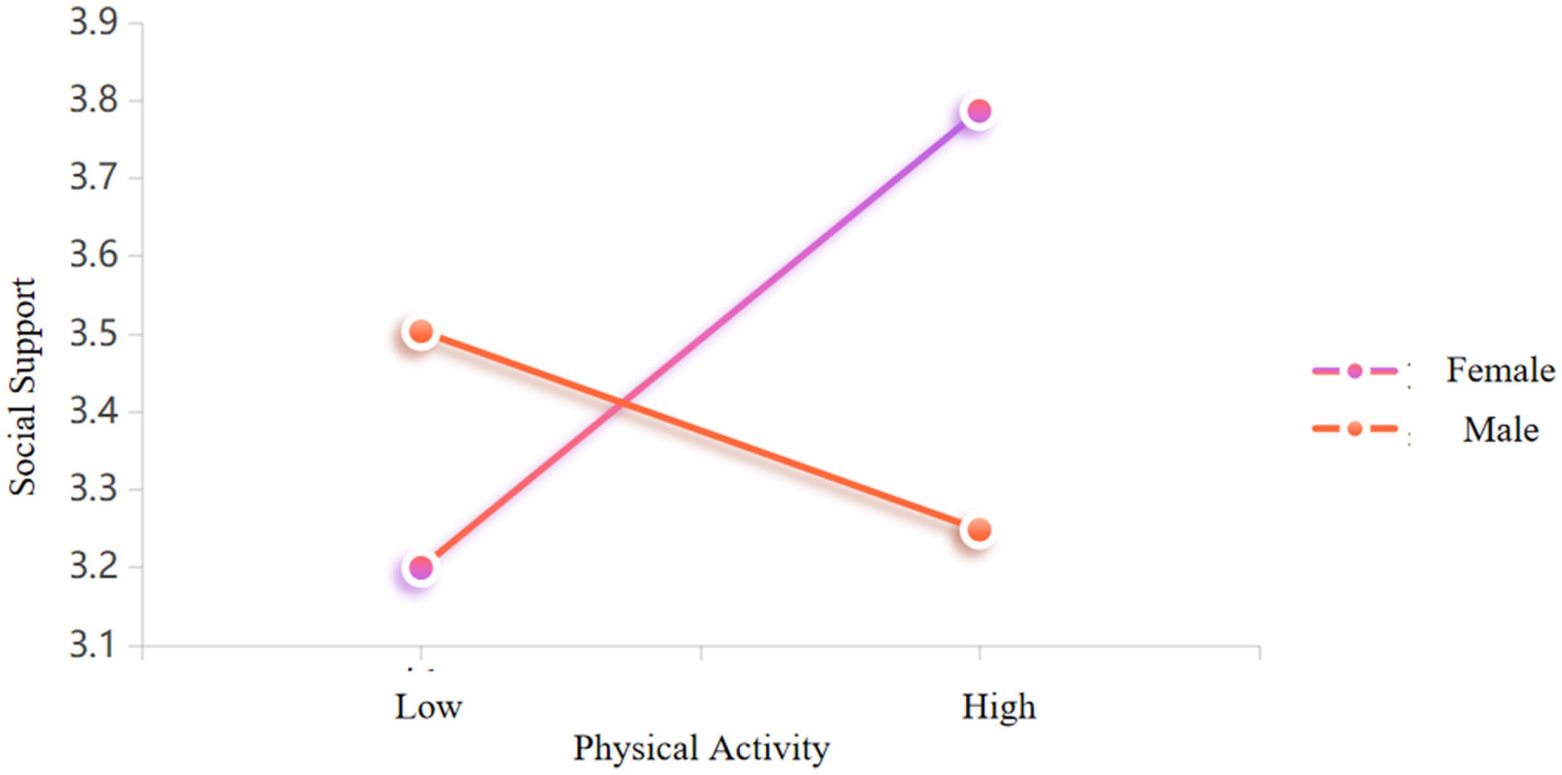
Simple slope analysis of gender.

To further probe the interaction mechanism between physical activity and gender, a simple slope analysis was conducted based on subgroup regression (see Table 4 and Figure 1). The results revealed a clear gender-specific pattern: Female college students (coded as 1): For every 1 standard deviation increase in physical activity, social support increased by 0.12 units (*β* = 0.12, SE = 0.01, *t* = 8.17, *p* < 0.001), with a 95% CI of [0.09, 0.15], which did not include zero, indicating a robust and significant positive predictive effect. Male college students (coded as 2): Physical activity was negatively associated with social support (*β* = −0.05, SE = 0.02, *t* = −3.22, *p* < 0.001), with a 95% CI of [−0.08, −0.02], also excluding zero, indicating a robust and significant negative effect. A comparison of effect magnitudes showed that the absolute value of the slope for females (0.12) was significantly greater than that for males (0.05), underscoring the substantive role of gender differences in shaping the impact of physical activity on social support (see Figure 3).

## Discussion

This study focused on Chinese college students and systematically examined the mechanism through which physical activity influences social support, with an emphasis on the mediating role of physical self-esteem and the moderating role of gender. The empirical findings demonstrated that physical activity indirectly enhances social support through improvements in physical self-esteem (indirect effect = 0.02, 95% CI [0.01, 0.03]), and that this pathway is significantly moderated by gender, producing a moderated mediation model that is contextually adaptive. These results not only deepen the understanding of the psychosocial functions of physical activity but also provide new empirical evidence for the applicability of social integration theory in contemporary college populations.

### Direct effects and the mediating mechanism

The study confirmed a significant positive effect of physical activity on social support (H1 supported), aligning with prior research and further substantiating the propositions of social integration theory (Lonsdale et al., 2013; Bem, 1972). According to this theory, collective activities create structured opportunities for social interaction, which in turn facilitate the development and strengthening of social networks (Scarapicchia et al., 2017). In the present context, team-based physical activities such as basketball or group dance classes serve as prototypical structured social environments. Within these settings, participants engage in shared goal achievement (e.g., completing training tasks), cooperative interaction (e.g., tactical coordination), and emotional bonding (e.g., post-game socializing), progressively building trust and friendship, and ultimately expanding and consolidating social support networks.

Beyond the direct association, the study revealed the partial mediating role of physical self-esteem (H3 supported), which accounted for one-third of the total effect. This finding advances the understanding of the “behavior → internal cognition → social function” transformation pathway. Drawing from self-perception theory (Hyde, 2005), consistent engagement in physical activity leads to observable improvements in physiological capacity (e.g., endurance), appearance (e.g., reduced body fat), or athletic performance (e.g., increased running speed). Through self-observation and attribution processes, individuals internalize these changes into a positive self-evaluation of their physical condition—enhancing physical self-esteem (Cohen and Wills, 1985). In turn, elevated physical self-esteem reduces social anxiety (e.g., fear of negative body evaluation), increases willingness to initiate interpersonal contact, and fosters greater participation in social activities (Bem, 1972). This enhanced social engagement facilitates the acquisition of emotional and instrumental support, thereby elevating overall perceived social support.

### The moderating role of gender

The most noteworthy finding of this study lies in the significant moderating effect of gender (H4 supported), which not only influenced the strength but also the direction of the physical activity–social support association. For female students, physical activity showed a significant positive association with social support (Eagly and Wood, 2012; Knight et al., 2011). This is consistent with the predictions of social role theory, which posits that female social roles emphasize affiliation, cooperation, and interpersonal harmony. The types of physical activity preferred by women (e.g., yoga, dance, group aerobics) often integrate elements of health promotion, body shaping, and social interaction. These activities align closely with socially prescribed female role expectations and inherently provide rich opportunities for emotional exchange and the formation of supportive relationships. Moreover, the self-confidence gained from improved physical self-esteem is readily transferred to socially congruent and proactive interaction patterns, further reinforcing the accumulation of social support resources.

For male students, however, the relationship was unexpectedly negative: higher levels of physical activity were associated with lower perceived social support. This finding challenges a straightforward application of social role theory and highlights the potential adverse consequences of hypercompetitive sport cultures (Eagly and Wood, 2012; Ekeland et al., 2002). In the male social role framework, competitiveness, dominance, and achievement are emphasized (Eagly and Wood, 2012). In the university context, male students often engage in high-intensity, competitive activities (e.g., competitive ball games, strength training) where the interaction climate is heavily outcome-oriented. Such environments may erode team cohesion, induce rivalry and exclusion, and hinder the formation of deep emotional connections. Additionally, intensive training schedules may displace other diverse social engagements (e.g., clubs, informal gatherings) that are essential for building broad and varied support networks (Ekeland et al., 2002). Meanwhile, the research logic of Ma et al. (2022) on Chinese team sport athletes is consistent with the findings of this study. Their study revealed that when the team environment overemphasizes competitive goals while neglecting emotional connections among members, athletes’ perception of social support significantly decreases (*β* = −0.19, *p* < 0.01). Additionally, 61.2% of male athletes engaged in high-intensity competitive training reported that “competitive pressure among peers undermines the willingness to help each other”—a result that highly aligns with the finding of this study that higher competitive participation among males corresponds to lower social support (Ma et al., 2022). Although the study by Shang et al. (2023) on Chinese college athletes did not directly focus on gender differences, it pointed out that “the motivational orientation of sports participation significantly affects social functioning,” specifically, when athletes take “competing to win” as their core motivation, their perception of social support becomes limited to an “performance evaluation-oriented” interaction model, rather than establishing in-depth emotional connections. This conclusion can indirectly support the result of this study that male college students in northern China (Hebei sample) experience decreased social support due to a competitive orientation (*β* = −0.05, *p* < 0.001). In other words, the regional consistency of motivational orientations (northern universities place greater emphasis on competitive performance) may have amplified the negative correlation effect (Shang et al., 2023). Consequently, while male students may gain some instrumental support from their sports teams, they may lose opportunities for emotional support and belonging, leading to an overall decline in perceived social support.

### Practical implications

The present findings carry important implications for optimizing mental health education and physical activity promotion in Chinese universities: Gender-Sensitive Physical Activity Strategies: Universities should avoid a “one-size-fits-all” approach to physical activity promotion. For female students, programs can emphasize diverse, socially engaging activities that leverage the positive social benefits of exercise. For male students, the overemphasis on competitiveness should be balanced by promoting cooperative, strategy-based, and recreational activities (e.g., ultimate frisbee, hiking, cooperative outdoor challenges) to mitigate the social resource displacement effect and foster genuine interpersonal bonds. Psychosocial Support for Male Athletes: Male students engaged in high-intensity competitive sports, particularly varsity athletes or those with athletic specializations, may require additional psychological support. Workshops focusing on balancing competition with cooperation, team cohesion building, interpersonal communication skills, and stress management in sports contexts could help reshape sports values, reinforcing the notion that athletics should promote not only physical performance but also mental well-being and social harmony.

### Limitations and future directions

Several limitations of this study should be acknowledged: Cross-Sectional Design: The cross-sectional nature of this study precludes causal inferences. Reverse causality cannot be ruled out (e.g., male students with lower social support may self-select into more individualized, competitive sports). Future research should adopt longitudinal or experimental designs to clarify dynamic causal relationships.

#### Self-report measures

Although measures to control common method bias were implemented during both the questionnaire design and data analysis phases, the measurement of key variables in this study still relied on self-reports. Moreover, relying solely on Harman’s single-factor test may not fully rule out the potential impact of method bias. Specifically, while Harman’s single-factor test has confirmed that there is no significant common method bias in this study, no pre-control measures were adopted during the questionnaire design phase (e.g., adjusting the order of questionnaire item descriptions, adding reverse-scored items, or including lie detection items). Additionally, the reliance on self-reported questionnaires may introduce social desirability bias and recall bias. The former refers to the possibility that participants may deliberately adjust their responses to conform to socially accepted behavioral norms; the latter may lead to data bias due to participants’ vague memories of past behaviors (e.g., frequency of physical activity). Combining self-reports with objective measurement methods (e.g., using accelerometers to record physical activity, or assessing social support through peer nomination or structured interviews) would further enhance the validity of the data.

#### Sample representativeness

The samples of this study were all drawn from universities in Jiangsu Province, and this sampling scope limits the generalizability of the study’s conclusions to other geographical regions and cultural contexts. For instance, there are differences in campus sports culture and students’ social interaction patterns across universities in different regions of China—for example, some universities in northern China place greater emphasis on competitive sports, while some in southern China focus more on recreational sports. Thus, conclusions based solely on samples from Jiangsu may not be fully applicable to these other regions. If future studies can expand the sample scope to include participants from different geographical regions (e.g., North China, South China, Northwest China, etc.) and different types of institutions (e.g., comprehensive universities, normal universities, sports universities), it will help improve the robustness and generalizability of the conclusions.

#### Future research could further explore

Experimental interventions comparing the effects of high-intensity competitive versus low-intensity cooperative activities on social support outcomes. Boundary conditions of the negative association in male students, such as exercise motivation (intrinsic vs. extrinsic) or personality traits (e.g., narcissism, agreeableness). Neuroscientific mechanisms using tools such as functional magnetic resonance imaging (fMRI) to investigate how physical self-esteem influences social cognition and support acquisition at the neural level.

## Conclusion

Based on a valid sample of 667 Chinese college students, this study constructed and empirically tested a moderated mediation model linking physical activity, physical self-esteem, and social support, with gender as a moderator. The main conclusions are as follows: (1) Physical activity significantly promotes social support. Higher frequency and intensity of physical activity were significant predictors of elevated perceived social support among college students, providing fresh empirical support for social integration theory. (2) Physical self-esteem mediates the physical activity–social support relationship. Physical activity not only directly enhanced social support but also indirectly contributed to its improvement by increasing individuals’ physical self-esteem. This mechanism illuminates the psychological process by which overt behavioral engagement translates into enhanced social functioning. (3) Gender moderates the pathway from physical activity to social support. Among female students, the pathway exhibited a stronger positive effect, whereas among male students, the relationship was negative—likely due to the competitive orientation of their sports participation and the displacement of broader social resources.

From a theoretical perspective, these findings extend the application boundaries of both social integration theory and self-perception theory by identifying physical self-esteem as a psychological mechanism and gender as a contextual boundary condition. From a practical perspective, the study underscores the importance of integrating gender sensitivity into the design of campus-based physical activity and mental health interventions, particularly by optimizing male students’ sports participation patterns and enhancing their access to supportive social networks. Although this study benefits from a relatively large sample size, robust theoretical modeling, and advanced statistical methods, certain limitations remain, including the cross-sectional design, reliance on self-reported measures, and limited geographical representation. Future research should adopt longitudinal and multi-method approaches to further elucidate the dynamic causal pathways and neuropsychological mechanisms linking physical activity and social support.

## Data Availability

The raw data supporting the conclusions of this article will be made available by the authors, without undue reservation.
